# *APOE* ε4 allele, along with *G206D*-*PSEN1* mutation, alters mitochondrial networks and their degradation in Alzheimer’s disease

**DOI:** 10.3389/fnagi.2023.1087072

**Published:** 2023-06-29

**Authors:** Irene Costa-Laparra, Elena Juárez-Escoto, Carlos Vicario, Rosario Moratalla, Patricia García-Sanz

**Affiliations:** ^1^Neurobiology of the Basal Ganglia Laboratory, Department of Functional Systems and Neurobiology, Instituto Cajal, Spanish National Research Council (CSIC), Madrid, Spain; ^2^Centro de Investigación Biomédica en Red Sobre Enfermedades Neurodegenerativas (CIBERNED), Instituto de Salud Carlos III, Madrid, Spain; ^3^Stem Cells, Neurogenesis and Neurodegeneration Laboratory, Department of Molecular, Cellular and Developmental Neurobiology, Cajal Institute, Spanish National Research Council (CSIC), Madrid, Spain

**Keywords:** mitochondria, neurodegeneration, Alzheimer’s disease, autophagy, oxidative stress, lysosomes and mitochondria imaging

## Abstract

**Introduction:**

Alzheimer’s disease remains the most common neurodegenerative disorder, depicted mainly by memory loss and the presence in the brain of senile plaques and neurofibrillary tangles. This disease is related to several cellular alterations like the loss of synapses, neuronal death, disruption of lipid homeostasis, mitochondrial fragmentation, or raised oxidative stress. Notably, changes in the autophagic pathway have turned out to be a key factor in the early development of the disease. The aim of this research is to determine the impact of the *APOE* allele ε4 and *G206D-PSEN1* on the underlying mechanisms of Alzheimer’s disease.

**Methods:**

Fibroblasts from Alzheimer’s patients with *APOE* 3/4 + *G206D-PSEN1* mutation and homozygous *APOE* ε4 were used to study the effects of *APOE* polymorphism and *PSEN1* mutation on the autophagy pathway, mitochondrial network fragmentation, superoxide anion levels, lysosome clustering, and p62/SQSTM1 levels.

**Results:**

We observed that the *APOE* allele ε4 in homozygosis induces mitochondrial network fragmentation that correlates with an increased colocalization with p62/SQSTM1, probably due to an inefficient autophagy. Moreover, *G206D-PSEN1* mutation causes an impairment of the integrity of mitochondrial networks, triggering high superoxide anion levels and thus making *APOE* 3/4 + *PSEN1* fibroblasts more vulnerable to cell death induced by oxidative stress. Of note, *PSEN1* mutation induces accumulation and clustering of lysosomes that, along with an increase of global p62/SQSTM1, could compromise lysosomal function and, ultimately, its degradation.

**Conclusion:**

The findings suggest that all these modifications could eventually contribute to the neuronal degeneration that underlies the pathogenesis of Alzheimer’s disease. Further research in this area may help to develop targeted therapies for the treatment of Alzheimer’s disease.

## 1. Introduction

Alzheimer’s disease (AD) is a progressive neurodegenerative disorder marked by impaired behavior, cognitive dysfunction, and episodic memory loss; being the most common dementia in the elderly and the most prevalent neurodegenerative disease (Masters et al., 2015). From the genetic standpoint, AD can be divided into two different categories: the familiar form (<10% of all cases) and the sporadic form (90–95% of cases). The familial form (FAD) has an early onset. It is triggered by mutations in any of the three primary genes linked to AD: APP (amyloid precursor protein) gene, *PSEN1* (presenilin) or *PSEN2* (presenilin) gene (Dorszewska et al., 2016) in chromosomes 21 and 14, respectively. These genes contribute to the amyloidogenic pathway by which the APP protein is consecutively cleaved and processed to produce Aβ oligomers susceptible to aggregate (Hardy and Selkoe, 2002). On the other hand, sporadic AD (SAD) results from a complex mixture of genetic and environmental factors. However, its pathobiology is still under investigation (Dorszewska et al., 2016).

The allele ε4 of the *APOE* gene encoding the apolipoprotein E (APOE) is the major genetic risk factor for AD (Fernández-Calle et al., 2022). Within the central nervous system, astrocytes mainly produce this protein. It is critical in shuttling cholesterol to neurons to maintain cell membranes and synapses and allow their reparation after injury (Belloy et al., 2019; García-Sanz et al., 2021). In humans, there are three *APOE* isoforms, which differ only by 1 or 2 amino acids: *APOE2*, *APOE3*, and *APOE4*. The ε3 allele has the highest prevalence (78%), followed by ε4 (15%) and finally ε2 (7%) (Masters et al., 2015). The ε4 allele in homozygosis raises the risk of developing AD by twelve times (Heffernan et al., 2016). The ε4 allele is linked to an earlier onset of the disease (Khachaturian et al., 2004) and more severe cognitive impairment (Najm et al., 2019). It has an approximately 50% contribution to the development of SAD (Ashford, 2004). Furthermore, the presence of the ε4 allele is related to the appearance of amyloid-β (Ab) aggregates, the hyperphosphorylation of tau, and the disorganization of mitochondrial networks (Mahley et al., 2007; Cheng and Bai, 2018) and ultimately, it may account for specific phenotypic heterogeneity in AD (Emrani et al., 2020).

The senile plaques composed of extracellular Aβ aggregates and neurofibrillary tangles formed by hyperphosphorylated tau protein remain AD’s two most critical histopathological hallmarks (Lane et al., 2018; Vidal and Zhang, 2021). Growing evidence suggests that abnormal mitochondrial function is involved in AD pathophysiology (Castora et al., 2022). Environmental toxins, such as pesticides, heavy metals, and industrial waste products, can impair mitochondrial function and produce reactive oxygen species (ROS) and oxidative stress, which can damage neurons and contribute to AD pathogenesis (Sharma et al., 2021). Mitochondrial dysfunction is observed in AD subjects within the brain and systemically (Strope and Wilkins, 2023). AD is depicted by disrupted energy metabolism in the brain and increased levels of oxidative stress (Wang et al., 2020). Furthermore, Aβ aggregates may also destabilize Ca^2+^ homeostasis generating a Ca^2+^ overload in the mitochondria and forming a permeability transition pore in the inner mitochondrial membrane (Wacquier et al., 2020). Eventually, this event may trigger the release of cytochrome C from mitochondria and the collapse of the mitochondrial membrane potential (Brookes et al., 2004; Calvo-Rodriguez and Bacskai, 2021). Moreover, Ca^2+^ can increase the number of reactive oxygen species (ROS) through two mechanisms: nitric oxide production, which inhibits the mitochondrial IV complex, and the increase in the activity of the electron transport chain, which results in increased ROS production (Brookes et al., 2004; Calvo-Rodriguez and Bacskai, 2021). Indeed, defective mitophagy mediated a preserved mechanism of memory loss across the AD models (Xie et al., 2022; Zeng et al., 2022). The impairment of the endocytic, autophagic, and lysosomal pathways is considered initiated at the early stages of AD. It appears to be involved in most AD cases (Krance et al., 2022) and other neurodegenerative diseases (García-Sanz et al., 2018). Autophagy appears to be compromised in not only AD but also in other neurodegenerative disorders. Autophagy is essential in maintaining normal cell function by removing potentially harmful materials, including damaged organelles (such as mitochondria) and poorly folded or aggregated proteins (Vegh et al., 2019; Zeng et al., 2022). Defects in this mechanism lead to an accumulation of these toxic materials, eventually causing neuronal death as it occurs in some neurodegenerative diseases, including AD (Filippone et al., 2022; Griffey and Yamamoto, 2022).

The initiation of the autophagy pathway is regulated by a plethora of different proteins, the most remarkable of which are the mTOR and ULK complexes. In the presence of nutrients, mTOR is phosphorylated and inhibits autophagy through the phosphorylation (at specific inhibition sites) of ULK, which is one of the proteins that contribute to the initiation of the phagophore formation process. On the contrary, in nutrient deprivation situations, mTOR will stop inhibiting ULK so that phagophore formation can occur and, therefore, autophagy will be enhanced (Alers et al., 2012). In AD, mTOR and other proteins involved in autophagosome formation are especially susceptible to changes in their function due to oxidative stress (Lee et al., 2012; Filippone et al., 2022). Altered autophagy is broadly established in AD, leading to damaged organelles buildup, including mitochondria (Frake et al., 2015). However, current research remains controversial regarding which stages of autophagy are specifically impaired. A comprehensive assessment of the autophagic process in CA1 pyramidal hippocampal neurons from early and late-stage AD patients showed a remarkable upregulation of autophagy-related genes, reflecting increases in both autophagosome and lysosome biogenesis. This induced autophagy status appears to be an early mechanism response, and autophagy flux is gradually hampered due to the failure of the lysosomal degradation (Bordi et al., 2016).

In fact, with age and under stress, lysosomes accumulate lipofuscin, which cannot be degraded, leading to lysosomal dysfunction (Brunk and Terman, 2002; Trigo et al., 2022). Strikingly, in various lysosomal storage diseases, lysosomal defects initially produce a burden of amyloidogenic proteins (Monaco and Fraldi, 2020; Riera-Tur et al., 2022). In AD, allele ε4 of the *APOE* gene has been related to alterations in the endocytic, autophagic, and lysosomal processes (Schmukler et al., 2018; Eran and Ronit, 2022; Fernández-Calle et al., 2022). In addition, it has been shown that *PSEN1* mutations produce lysosomal and autophagic dysfunction due to defects in lysosomal acidification and lysosomal Ca^2+^ homeostasis (Coen et al., 2012; Lee et al., 2015; Yang et al., 2019; McDaid et al., 2020).

Overall, AD pathology generates increased oxidative stress and alterations in autophagy that compromise cellular homeostasis, favoring the mechanisms of neurodegeneration. In this study, we evaluate the impact of the presence of the *APOE*ε4 allele and a mutation in *PSEN1* over cellular viability, ROS production, mitochondrial structure, autophagy status, and lysosome accumulation and distribution in age-matched fibroblasts from healthy and AD patients. We used fibroblasts because they are easily isolated from skin biopsies preserving the chronological and biological aging of patients and their environment etiopathology. Indeed, they are extensively used as the model of several neurodegenerative disorders, including AD (Pani et al., 2009; Theendakara et al., 2016; Pérez et al., 2017; Olesen et al., 2022).

## 2. Materials and methods

### 2.1. Fibroblasts culture

Skin fibroblasts were generated from 6 AD patients with different allelic combinations of *APOE* (one also presenting a the G206D mutation in *PSEN1*) and six age-matched healthy controls (Table 1). The patients and control subjects were recruited and signed informed consent, previously accepted by the Human Research Ethics Committees Ethics of Spanish National Research Council (CSIC) and CIBERNED (Instituto de Salud Carlos III). All samples were sequenced at the laboratory of Dr. Joan Comella at the Hospital Vall d’Hebron (Lonza, Barcelona, Spain), according to the protocol established by Calero et al. (2009). Fibroblasts were maintained in DMEM (Lonza, Barcelona, Spain) with 10% FBS (Life Technologies, Alcobendas, Spain), 1% penicillin-streptomycin (Lonza, Barcelona, Spain), and 0.1% amphotericin B (Invitrogen, Madrid, Spain). In oxidative stress induction experiments, fibroblasts were treated with tert-Butyl hydroperoxide (tBHP, Luperox^®^ TBH70X, Sigma Aldrich, Madrid, Spain) at different concentrations. Experiments were conducted in all fibroblasts in parallel.

**TABLE 1:** Features of recruited patients and control subjects used in this study.

Patient (ID)	Gender	Age (years)	Genotype	Clinical features
**Alzheimer’s disease patients**				
AD1	M	74	*APOE* 4/4	Dementia (more severe symptoms than those of APOE3/3 patients)
AD2	F	66	*APOE* 4/4	Dementia, aphasia (more severe symptoms than those of APOE3/3 patients)
AD3	M	79	*APOE* 3/3	Loss of memory and prefrontal functions.
AD4	M	73	*APOE* 3/3	Dementia
AD5	F	79	*APOE* 3/3	Dementia
AD6	M	43	*APOE* 3/3 + mutation in *PSEN1*	Severe dementia, loss of memory, and aphasia.
**Control**				
C1	M	85	*APOE* 2/3	–
C2	F	66	*APOE* 3/3	–
C3	M	72	*APOE* 3/3	–
C4	M	72	*APOE* 2/3	–
C5	F	63	*APOE* 2/2	–
C6	F	71	*APOE* 3/3	–

### 2.2. Resazurin cell viability assay

We assessed cell viability using the resazurin assay, a fluorometric method to estimate cell metabolic activity. Only viable cells with healthy mitochondria can reduce non-fluorescent resazurin to resorufin (λemission = 585 nm) thanks to the electrons transferred by mitochondrial enzymes. In contrast, non-viable cells cannot perform this reduction and do not spawn a fluorescent signal. Fibroblasts were seeded at 62,500 cells/cm^2^ in a MW96 plate, allowed to proliferate for 24 h, and then incubated with different treatments. After that, resazurin (Sigma-Aldrich, Madrid, Spain) was added at 40 μg/μl, shaken for 1–2 min, and incubated in darkness for 30 min at 37°C, 5% CO_2_. Finally, the fluorescence emission signal (585 nm) was detected with the plate reader FLUOstar Omega (BMG LABTECH, Allmendgrün, Germany).

### 2.3. Flow cytometry

A superoxide anion was detected using the fluorescent probe dihydroethidium (DHE, Invitrogen, Madrid, Spain) to measure the intracellular ROS content. Fibroblasts were seeded in MW6 plates at 20,800 cells/cm^2^, allowed to proliferate for 24 h, and then incubated with tBHP 300 μM for 1 h. After that, cells were trypsinized, pelleted by centrifugation, and incubated in the darkness for 30 min at 37°C with DHE 1 μM. Finally, cells were centrifuged again and resuspended in PBS 1X. Twenty thousand events were acquired with CytoFLEX Flow Cytometer (Beckman Coulter, Krefeld, Germany).

### 2.4. Western blot

Western Blot experiments were performed with cell lysates of the fibroblasts under basal conditions and after a 4 h treatment with EBSS (Earle’s Balanced Salt Solution, Sigma-Aldrich). For LC3-II western blot, cells were treated with chloroquine (CQ) 50 μM 4 h. Cells were lysed in lysis buffer (50 mM Tris HCl pH 7.4, 1 mM DTT, 20 mM β-Glycerophosphate, Triton X-100). Supernatants were obtained after a 30 min centrifugation, and the protein concentration was quantified with the BCA Assay Kit (Sigma). 5–12 μg of protein lysates were loaded onto a SDS-electrophoresis gel and then transferred to nitrocellulose membranes. The membranes were blocked with appropriate 5–10% BSA or skim milk. Then, they were incubated with primary antibodies for TOM20 (Santa Cruz Biotechnology, Heidelberg, Germany, 1:1000 dilution), p-mTOR Ser2448 (Cell Signaling Technology, Leiden, Netherlands, 1:1000), mTOR (Cell Signaling Technology, Leiden, Netherlands, 1:1000), p-ULK Ser757 (Cell Signaling Technology, Leiden, Netherlands, 1:1000), ULK (Cell Signaling Technology, Leiden, Netherlands, 1:1000), Beclin-1 (Santa Cruz Biotechnology, Heidelberg, Germany, 1:1000), LC3-II (Sigma, 1:5000). Actin (Sigma-Aldrich, 1:30000) and β-tubulin (Cell Signaling Technology, Leiden, Netherlands, 1:20000) were used as the loading control. The appropriate secondary infrared dye-conjugated antibodies (α-mouse IRDye 800 CW and α-rabbit IRDye 680 LT, LI-COR Biosciences, Lincoln, NE, United States, 1:15000) were detected by Odyssey Infrared Imaging System (LI-COR Biosciences, Lincoln, NE, United States). For each assay, a minimum of 3 independent experiments were carried out.

### 2.5. mtDNA content

Total DNA was isolated from fibroblasts using the Quick-DNA Miniprep Plus Kit (Zymo Research, Irvine, CA, United States), following the manufacturer’s instructions. The Mitochondrial DNA (mtDNA) and the nuclear DNA (nDNA) content were determined by using specific primers for the mitochondrial tRNA^Leu(UUR)^ and 16S rRNA genes and for the nuclear β-2-microglobulin (β2M) gene, respectively (Venegas et al., 2011). Quantitative PCR was carried out with SYBR Green Master Mix (Applied Biosystems, Alcobendas, Spain), and the fluorescence amplification cycles were used to calculate the mtDNA: nDNA ratio for each sample.

### 2.6. Immunocytochemistry

Fibroblasts (7,400 cells/cm^2^) were seeded on gelatin-coated round glass coverslips (12 mm) in MW24 plates and subsequently fixed with 4% paraformaldehyde or methanol, as appropriate. Immunohistochemistry was done as previously described (Ruiz-DeDiego et al., 2015). After a 1 h blocking step with 10% BSA/0.1% Triton/PBS, fibroblasts were incubated with primary antibodies for p62/SQSTM1 (Progen, Heidelberg, Germany, 1:200) and LAMP1 (Santa Cruz Biotechnology, Heidelberg, Germany, 1:300). Afterward, fibroblasts were incubated with secondary antibodies conjugated to Alexa Fluor 594 or 488. Fibroblasts were counterstained with DAPI (Thermo Fisher Scientific, Madrid, Spain). Finally, coverslips were mounted with Prolong^®^ Gold (Life Technologies, Alcobendas, Spain). Images were acquired with a SP5 laser confocal microscope (Leica, Wetzlar, Germany).

### 2.7. LysoTracker and Filipin staining

Fibroblasts were seeded in round gelatin-coated glass coverslips at a density of 7,400 cells/cm^2^. To label the lysosomes, fibroblasts were incubated with 70 nM lysoTracker Red DND-99 probe (Invitrogen, Madrid, Spain; λem = 590 nm) for 30 min at 37°C. Next, fibroblasts were fixed with 3% paraformaldehyde (PFA) for 30 min at RT, washed with glycine and stained with 25 μg/ml Filipin (Sigma; λem = 400–484 nm) for free cholesterol detection. Finally, the round covers were mounted with Prolong^®^ Gold reagent (Life Technologies, Alcobendas, Spain) and observed using the fluorescence microscope (Leica, Wetzlar, Germany).

### 2.8. Lentivirus production

A specific lentivirus was used to analyze the complexity of the fibroblasts’ mitochondrial networks of fibroblasts. Specifically, we used a plasmid with mtDsRed red fluorescent protein (pWPXL-mtDsRed, λex = 580 nm/λem 630/60 nm; Clayton et al., 2012), kindly provided by Dr. Ramón Trullas, from the Instituto de Investigaciones Biomédicas of Barcelona. Constructions in the pWPXL lentiviral vector contain a target sequence of the subunit IV of the mitochondrial protein cytochrome oxidase. HEK293T cells were used as packaging cells to obtain these lentiviruses. These cells contain the SV40 virus T antigen, which allows an episomal replication of plasmids containing the origin of replication of this virus. The fibroblasts were seeded at 70,500 cells/cm^2^ in 100 mm plates and transfected with the following plasmid mix: pMD2.G (viral envelope), psPAX2 (viral capsid), and pWPXL-mtDsRed in a 1:2:3 ratio, using calcium chloride (CaCl_2_). Lentiviral particles were collected twice: 8 h and 16 h after removing the transfection medium. Finally, the medium containing the lentiviral particles was ultracentrifuged at 20,000 rpm, and 16°C for 2 h and the pellet was resuspended in PBS and tittered by qPCR.

### 2.9. Fibroblast infection

Fibroblasts of all genotypes were seeded in round gelatin-coated glass coverslips (2,000 cells/cm^2^) and transduced with the lentiviral particles containing the plasmid pWPXL-mtDsRed to obtain 43 integrations/cell. Cells were fixed with 4% paraformaldehyde 36 h later and used for p62/SQSTM1 immunocytochemistry. Finally, we mounted coverslips with fibroblasts using Prolong Gold (Thermo Fisher Scientific, Madrid, Spain). Images were captured and assessed with a SP5 laser confocal microscope (Leica, Wetzlar, Germany).

### 2.10. Image analysis

All images were acquired with an SP5 laser confocal microscope (Leica, Wetzlar, Germany), using the 63X objective and 3.5X digital zoom. For images of the mitochondrial structure, z-stacks of 6 confocal images were obtained, separated by a vertical distance of 0.5 μm. Maximum projections of the images were analyzed with FIJI-ImageJ software [National Institutes of Health (NIH), Bethesda, MD], using the MiNA plug-in (Valente et al., 2017) to obtain data related to different parameters of the mitochondrial networks (number of individuals and networks, mean of branch length and network size, and mitochondrial footprint). The area analyzed in each image corresponds to a region of interest (ROI) of 150 × 150 pixels located in a perinuclear region of the cell. At least 6 cells per subject were examined.

The following analysis was carried out using custom-written scripts of the FIJI-ImageJ developed by the Scientific Image and Microscopy Unit of Cajal Institute: (i) Colocalization between p62/SQSTM1 and mitochondria using Manders’ Coefficients. Colocalization depicted the spatial superimpose of signal intensities from isolated image channels. The Manders’ Coefficients, tM1 and tM2, reflect the degree of bidirectional colocalization between two images. The tM1 coefficient refers to the sum of signal intensities in Channel 1 having corresponding components in Channel 2, divided by the sum of total intensities in Channel 1. In this case, p62/SQSTM1 is depicted in Channel 1 (green), and mitochondria belong to Channel 2 (red). Thus, tM1 ultimately represents the quantification of mitochondria labeled with p62/SQSTM1. The tM2 coefficient is similarly computed, the sum of signal intensities in Channel 2 having corresponding factors in Channel 1, split by the sum of total intensities in Channel 2. In other words, tM2 represents the percentage of p62/SQSTM1 labeled mitochondria. (ii) Quantifying the fluorescence integrated density (IntDen) of p62/SQSTM1, LAMP1, and LysoTracker. Briefly, images were converted to grayscale, their background was subtracted, and the contour of each cell was drawn to obtain the value of its area. Finally, the fluorescence signal was set to a threshold to determine the integrated intensity values (IntDen; the intensity of the fluorescence signal divided by the total cell area). (iii) Analysis of the distribution of lysosomes (LAMP1). Maximum projections of full cell thickness z-stacks were obtained, separated by a vertical distance of 0.6 μm. Then, confocal images were turned into binary ones to visualize the LAMP1 positive area inside each cell and differentiate between individual and grouped lysosomes (Individuals = area < 1.3 μm and circularity > 0.6; Groups = area ≥ 1.3 μm and circularity ≤ 0.6). The lysosome clustering index was calculated as the Groups/Individuals ratio. At least 6 cells were analyzed for each subject in these three analyses. (iv) The proportion of fibroblasts presenting the perinuclear lysosomal clustering phenotype was also quantified using ImageJ software. This phenotype was analyzed based on previous studies (Hockey et al., 2014; García-Sanz et al., 2017). We considered cells positive for the clustering phenotype if they presented highly packed perinuclear lysosomal aggregates and negative if they presented lysosomes uniformly distributed in the perinuclear region or the whole cell. (v) To quantify Filipin in LysoTracker-positive-tagged lysosomes, we overlapped a LysoTracker mask over Filipin images to calculate Filipin IntDen as described (García-Sanz et al., 2017).

### 2.11. Statistical analysis

We performed at least 3 independent experiments per assay to obtain all data. We normalized the data acquired to control values as appropriate. The statistical analysis of the results was carried out with GraphPad Prism 6.0 (Graphpad software, La Jolla, CA, United States). Data distribution was evaluated using the D’Agostino and Pearson test. One or two-way ANOVA parametric tests were used, followed by the *post-hoc* Bonferroni, to compare results between different fibroblast lines and treatments. In cases where the data had a non-Gaussian distribution, we applied the Kruskal-Wallis test and Dunn’s *post-hoc*. Statistical significance was set at a *p*-value of *P* < 0.05.

## 3. Results

### 3.1. *APOE* 3/4 genotype in combination with *PSEN1* mutation is prone to cell vulnerability induced by oxidative stress

**FIGURE 1:**
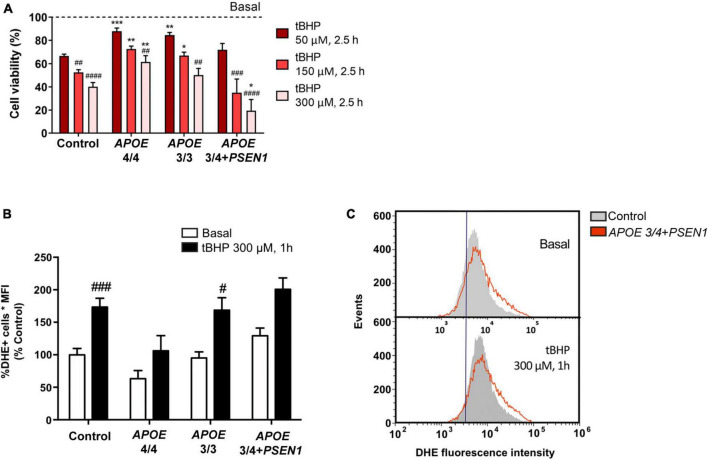
Vulnerability of fibroblasts to tert-butyl (tBHP) induced oxidative stress and increased production of ROS species in *APOE* 3/4 + *PSEN1* fibroblasts. **(A)** Percentage of the viability of fibroblasts from AD patients compared to controls and to respective basal conditions. **(B)** Quantification of DHE signal measured as the percentage of DHE positive cells by the mean fluorescence intensity in the fibroblasts under basal conditions and after 1 h treatment with 300 μM tBHP. **(C)** Representative graph of events versus fluorescence intensity of the DHE probe obtained by flow cytometry from control and *APOE* 3/4 + *PSEN1* fibroblasts. Samples were processed in parallel; data represent the mean ± SEM of at least *n* = 3 independent experiments for all cell lines. *P < 0.05; **P < 0.01; ***P < 0.005 vs. Control; ^#^*P* < 0.05; ^##^*P* < 0.01; ^###^*P* < 0.005; ^####^*P* < 0.0001 vs. basal; following 2-way ANOVA, *post hoc* Bonferroni.

To evaluate whether the ε4 or ε3 allele of the *APOE* gene and/or the *PSEN1* mutation could contribute to cell vulnerability, we performed resazurin-based cell viability tests in control and AD fibroblasts. Oxidative stress was induced by tert-butyl hydroperoxide (tBHP). We found that this treatment significantly decreased cell viability in all fibroblasts, regardless of their genotype. This decrease was proportional to the concentration of tBHP used in the treatment: 50 μM, 150 μM, and 300 μM, during 2.5 h (Figure 1A). However, *APOE 4/4* and *APOE 3/3* fibroblasts showed a significantly smaller decrease in cell viability compared to controls. While *APOE 3/4* + *PSEN1* fibroblasts showed a significantly higher decrease in viability, particularly at 300 μM, indicating that they are more vulnerable to the treatment than controls. To determine whether these viability alterations affect the cellular redox state, we performed a flow cytometry assay using the dihydroethidium (DHE) probe in all fibroblasts. This assay allowed us to detect the superoxide anion levels after treatment with tBHP for 1 h at 300 μM (the concentration which produces the most significant changes in cell viability). As expected, tBHP increased superoxide anion in all fibroblasts (Figure 1B). *APOE 3/4* + *PSEN1* fibroblasts show slightly higher superoxide anion levels than controls at baseline and after treatment with tBHP, although this difference is not statistically significant (Figures 1B, C). In summary, fibroblasts carrying the *PSEN1* mutation show decreased viability, thus more vulnerable to oxidative stress. In contrast, *APOE 4/4* and *APOE 3/3* genotypes appear to be more resistant to oxidative stress.

### 3.2. Lysosomal free cholesterol is impaired in *APOE 3/4 + PSEN1* fibroblasts

**FIGURE 2:**
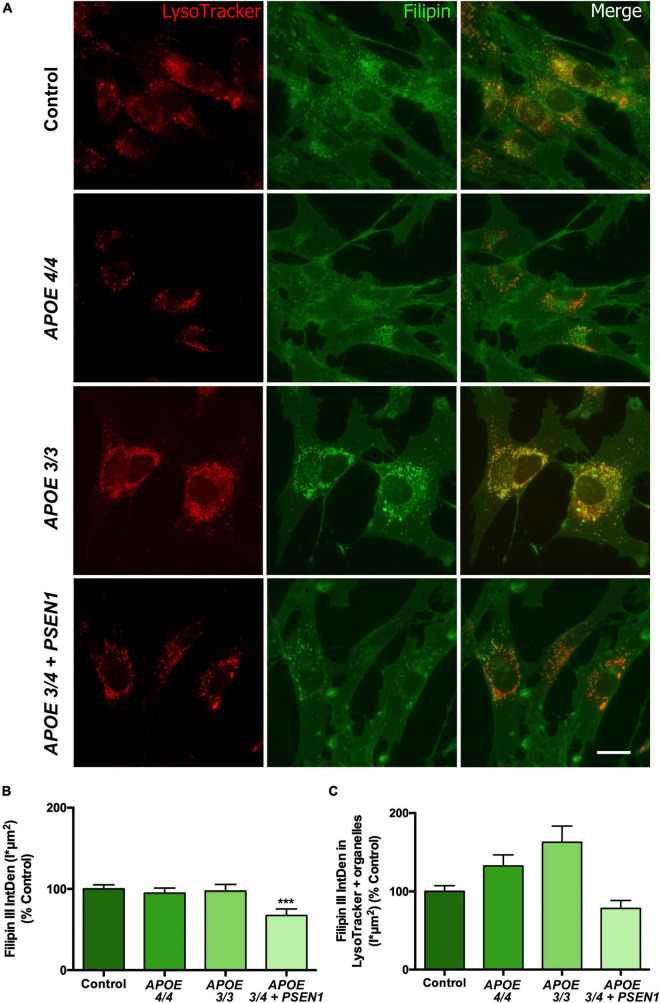
Representative images and quantification of total free cholesterol and free cholesterol levels inside the lysosomes of control and AD fibroblasts. **(A)** Representative confocal images of control and AD fibroblasts stained with LysoTracker (acid compartments, red) and Filipin (free cholesterol, green). **(B)** Integrated density (IntDen = intensity*μm2) quantification of Filipin in the total cell area. **(C)** Integrated density (IntDen = intensity*μm2) quantifications of Filipin in the area occupied by LysoTracker staining. Samples were processed in parallel; data represent mean ± SEM of *n* = 2 independent experiments, with a minimum of 80 cells per genotype analyzed. ***P < 0.005 vs. control by Kruskal Wallis, *post-hoc* Dunn. Calibration bar = 20 μm.

Several studies underscore that alterations in cholesterol metabolism are involved in the pathogenesis of AD (Wood et al., 2014; Loera-Valencia et al., 2019; van der Kant et al., 2019), and APOE mediates cholesterol exchange between brain cells (Liu et al., 2013). Thus, we next measured free cholesterol levels using Filipin staining in combination with the LysoTracker probe. Filipin staining of all fibroblasts disclosed intracellular punctuate structures. In addition, these structures were identified as lysosomes according to co-labeling with the LysoTracker probe (Figure 2). Total free cholesterol levels (quantified by Filipin IntDen; García-Sanz et al., 2017; Wilhelm et al., 2019) were significantly decreased in APOE 3/4 + PSEN1 fibroblasts. In contrast, APOE 3/3 and APOE 4/4 fibroblast did not show significant changes compared to controls (Figures 2A, B). Next, we quantified free cholesterol in lysosomes. Strikingly, we found that Filipin IntDen in LysoTracker-positive organelles is higher, but not significant, in both APOE 3/3 and APOE 4/4 fibroblasts than in controls, while in APOE 3/4 + PSEN1 fibroblasts the levels are again slightly decreased (Figures 2A, C).

### 3.3. Aberrant mitochondrial networks in AD patients with *APOE 4/4 and APOE 3/4 + PSEN1* genotypes

**FIGURE 3:**
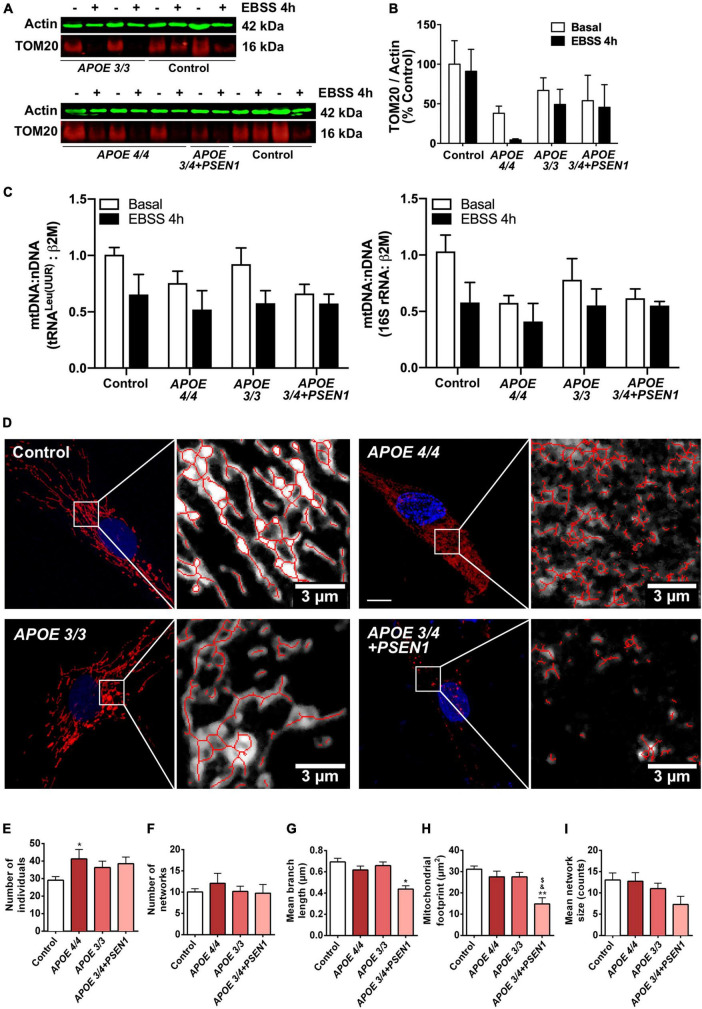
Alterations in mitochondrial morphology in fibroblasts of AD patients. **(A)** Representative western blots of TOM20 levels after treatment with EBSS for 4 h. **(B)** Quantitative densitometry of TOM20. Samples were processed in parallel; data represent mean ± SEM of *n* = 3 independent experiments. 2-way ANOVA, *post-hoc* Bonferroni. **(C)** Normalized mtDNA:nDNA ratio, calculated with the levels of the mtDNA genes tRNA^Leu(UUR)^ (left) and 16S rRNA (right) after treatment with EBSS for 4 h. Data represent mean ± SEM of *n* = 3 independent experiments. **(D)** Representative confocal images of mtDsRed stained mitochondria in control and AD fibroblasts and examples of the skeletonization of the networks obtained with the MiNA plug-in for ImageJ. Nuclei are stained with DAPI. Calibration bar = 10 μm. **(E–I)** Analysis of different parameters related to the complexity of the mitochondrial networks. Samples were processed in parallel; data represent mean ± SEM of *n* = 6 images for all cell lines. *P < 0.05; **P < 0.01 vs. Control; ^$^*P* < 0.05 vs. *APOE*3/3; ^&^*P* < 0.05 vs. *APOE*4/4 by One-way ANOVA, *post-hoc* Bonferroni.

Since the altered resazurin reduction and the slight increment in ROS production in some of the AD fibroblasts could be due to mitochondrial dysfunction, we next analyzed the status of mitochondria in AD fibroblasts. First, the levels of the mitochondrial internal membrane protein TOM20 (a marker for mitochondrial biomass) were measured by Western blot (Figures 3A, B). At baseline, AD fibroblasts showed a slight, though not significant, decrease of TOM20 compared to controls (Figure 3B). After 4 h-EBSS treatment to induce autophagy, only *APOE 4/4* fibroblasts displayed a reduction in TOM20, although not significant as before. In addition, we determined the mtDNA content in control and AD fibroblasts (Figure 3C). The levels of the mtDNA genes tRNA^Leu(UUR)^ and 16S rRNA were obtained by qPCR and normalized to a nuclear gene. Consistent with western blot results, all AD fibroblasts showed a slight non-significant decrease in basal mtDNA content compared to controls (Figure 3C).

Next, to determine mitochondrial morphology in greater detail, fibroblasts were infected with pWPXL-mtDsRed lentivirus to label mitochondrial networks. First, confocal images of the mitochondrial networks showed that control fibroblasts distribute their mitochondria as reticulum-like uninterrupted networks, spread through the cytoplasm (Ježek and Plecitá-Hlavatá, 2009), while AD fibroblasts present a less reticular distribution than controls (Figure 3D). In particular, mitochondria in *APOE 3/4* + *PSEN1* fibroblasts displayed a stippled appearance rather than the usual network one. Secondly, MiNA structural analysis (Valente et al., 2017; Figures 3E–I) showed a higher number of fragmented individual mitochondria in AD fibroblasts than in controls, which is only significant in the case of the *APOE 4/4* genotype (Figure 3E).

Remarkably, *APOE 3/4* + *PSEN1* genotype also produces a significant decline in the length of the mitochondrial branches (Figure 3G), in the cellular area occupied by mitochondria (mitochondrial footprint, Figure 3H) and in the size of the mitochondrial networks but not being significant in this latter case (Figure 3I). All these results suggest that the mitochondrial network’s morphology and integrity are compromised in all fibroblasts from AD patients and that these changes are much more evident in *APOE 3/4* + *PSEN1* fibroblasts and in *APOE 4/4*.

### 3.4. *APOE 4/4* fibroblasts increase mitochondria degradation via p62/SQSTM1

**FIGURE 4:**
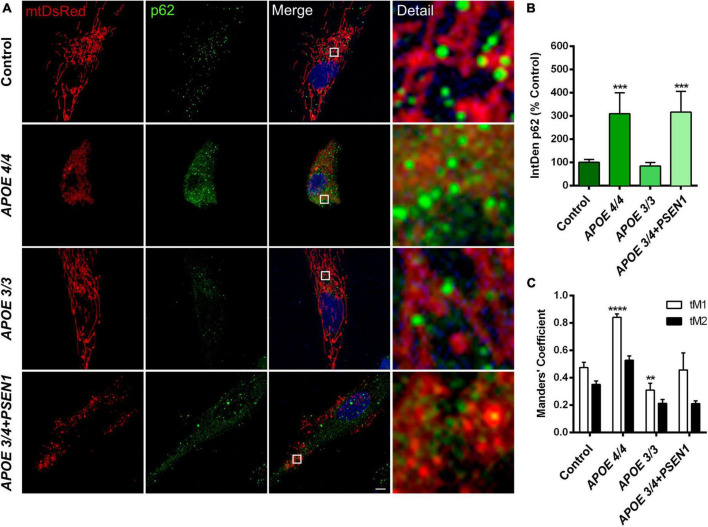
Mitochondria and p62/SQSTM1 colocalization in AD fibroblasts. **(A)** Representative images of mtDsRed-labeled mitochondria and immunofluorescence for p62/SQSTM1 in AD fibroblasts (*APOE* 4/4, *APOE* 3/3, and *APOE* 3/4 + *PSEN1*) Nuclei are stained with DAPI. **(B)** Integrated density (IntDen = intensity*μm2) quantifications of p62/SQSTM1. **(C)** Quantitation of mtDsRed and p62/SQSTM1 colocalization with Manders’ Coefficients (tM1 and tM2). Samples are processed in parallel; data represent mean ± SEM of *n* = 6 images for all cell lines. **P < 0.01; ***P < 0.005; ****P < 0.0001 vs. control by One-way ANOVA, *post-hoc* Bonferroni. Calibration bar = 10 μm.

Since mitophagy could be induced in AD fibroblasts to eliminate damaged mitochondria, specifically in those with the ε4 allele (*APOE* 4/4 and *APOE* 3/4+*PSEN1*), we first performed a p62/SQSTM1 immunofluorescence in all fibroblasts (Figure 4A). The quantification of the fluorescence integrated density (IntDen) of p62/SQSTM1 showed an increase in *APOE* 4/4 and *APOE* 3/4 + *PSEN1* fibroblasts (Figure 4B).

Subsequently, to determine if mitochondria are suitably targeted for degradation via mitophagy in AD fibroblasts, we labeled p62/SQSTM1 in fibroblasts infected with pWPXL-mtDsRed. We quantified the colocalization of p62/SQSTM1 with mitochondria (mtDsRed) using Mander’s Coefficients (tM1 and tM2). The results showed that, in all fibroblasts, the tM1 coefficient is always higher than tM2 (Figure 4C), which indicates that the percentage of mitochondria being labeled for degradation is higher than the percentage of p62/SQSTM1 that is intended to mark those mitochondria (that is, there is a certain percentage of p62/SQSTM1 that remains free to mark other substrates for degradation). In addition, we found that *APOE* 3/3 fibroblasts have significantly fewer mitochondria marked for degradation than controls, which is consistent with *APOE* 3/3 fibroblasts not showing significant changes in the structure of the mitochondrial networks compared to controls. In contrast, *APOE* 4/4 fibroblasts had a much significantly higher percentage of mitochondria that will probably undergo mitophagy (Figure 4C). These results correlate with the noted disruption of the mitochondrial networks found in *APOE* 4/4 fibroblasts (Figure 3E). However, the disruption of the mitochondrial networks shown by *APOE 3/4* + *PSEN1* fibroblasts did not correlate with increased colocalization of p62/SQSTM1 with mitochondria (Figure 4C). Thus, in this case, the morphological changes may not be due to an increased effective mitophagy.

### 3.5. Autophagic flux is slightly induced in AD fibroblasts

**FIGURE 5:**
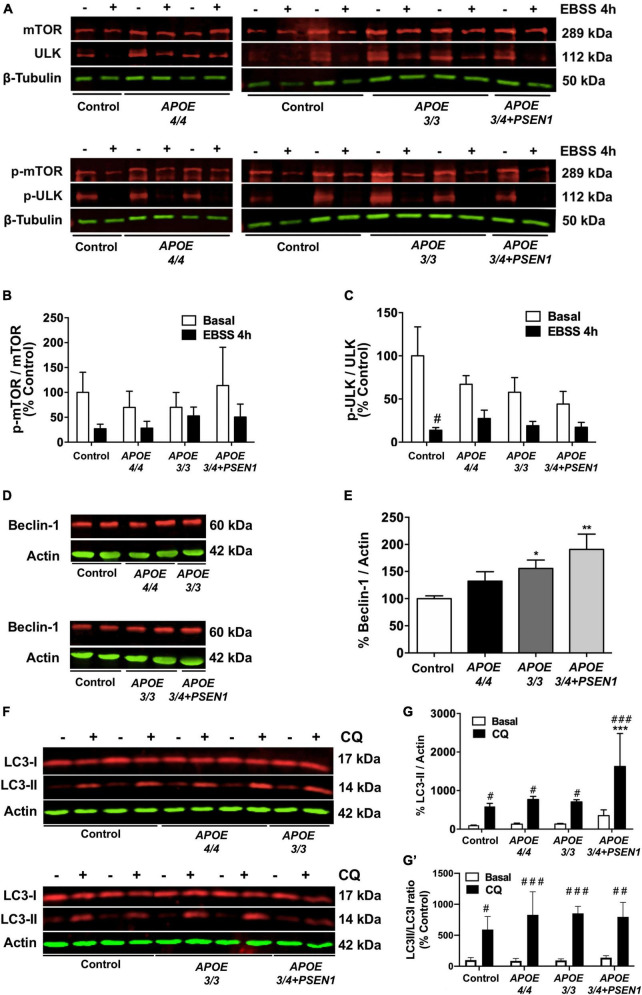
Impaired autophagy in AD fibroblasts. **(A)** Representative western blots of p-mTOR and p-ULK levels under baseline and nutrient deprivation conditions (EBSS treatment for 4 h). **(B)** Quantitative densitometry of p-mTOR (normalized against β-tubulin and total mTOR levels). **(C)** Quantitative densitometry of p-ULK (normalized against β-tubulin and total ULK levels). **(D)** Representative Western blot of Beclin-1 levels. **(E)** Quantitative densitometry of Beclin-1 (normalized against β-actin). **(F)** Representative Western blot of LC3-II and LC3I levels under baseline and chloroquine (CQ) treatment conditions. **(G)** Quantitative densitometry of LC3-II (normalized against β-actin). **(G′)** Quantitative densitometry of LC3II (normalized against LC3I) relative to the ratio from untreated controls. Samples are processed in parallel; data represent mean ± SEM of *n* = 3 independent experiments for all cell lines. *P < 0.05 vs. Control; **P < 0.01 vs. Control; ^#^*P* < 0.05 vs. basal; ^##^*P* < 0.01 vs. basal; ^###^*P* < 0.005 vs. basal. 2-way ANOVA, *post-hoc* Bonferroni [Kruskal Wallis, *post-hoc* Dunn for panel **(E)**].

To further investigate if the changes observed in the mitochondrial networks of the fibroblasts with the ε4 allele of the *APOE* gene are due to an increased mitophagy, we studied the autophagic pathway (Figure 5). The phosphorylation of mTOR, one of the central regulators of the pathway, and ULK, its target protein as well as Beclin-1 (an essential protein complex for the formation of the autophagosome) were determined by Western blot. Under basal conditions (in the presence of nutrients), mTOR is phosphorylated, and the p-mTOR-dependent phosphorylation of the initiator protein ULK inhibits autophagy. Thus, at baseline state, p-mTOR and p-ULK levels are increased. On the contrary, under nutrient deprivation conditions (4 h EBSS treatment), mTOR de-phosphorylates and stops inhibiting ULK, thus favoring the induction of autophagy. As expected, starvation decreased the levels of phosphorylation of both proteins in all fibroblasts (Figures 5A–C). However, under basal conditions, we also detected a slight (but not statistically significant) decrease in the mTOR and ULK phosphorylation in AD fibroblasts compared to controls, more evident in the case of p-ULK (Figure 5C). This could indicate that basal mTOR-dependent autophagy is moderately induced, which is consistent with our previous results for *APOE 4/4* fibroblasts showing a slight decrease in mitochondrial biomass and an increased colocalization with p62/SQSTM1. Likewise, Beclin1 was increased in AD fibroblasts under basal conditions, only significant in *APOE 3/3* and *APOE 3/4* + *PSEN1* fibroblasts (Figures 5D, E). These results suggest that AD fibroblasts can over-activate autophagy through mTOR- and Beclin-1-dependent mechanisms. Then, we assessed the lipidation of LC3I into LC3-II as a potential marker of autophagosome formation. Our Western blot results displayed that basal LC3-II was slightly increased, although not significant, in *APOE 3/4* + *PSEN1* fibroblasts over controls (Figures 5F, G). However, when we determined the LC3II/LC3I ratio, we found no differences in basal conditions. CQ treatment significantly increased LC3-II levels normalized to β-actin (Figures 5F, G) and increased LC3II/LC3I ratio compared to basal conditions in all fibroblasts (Figure 5G′). We suggest that the accumulation of LC3II is due to the inhibition of the autophagosome-lysosome fusion produced by CQ, which leads to hindering the regular degradation of LC3II rather than to a new process of lipidation. Moreover, we found that CQ significantly potentiated the LC3II buildup in the *APOE 3/4* + *PSEN1* fibroblasts compared to controls (Figures 5F, G) but did not enhance LC3 lipidation measured by LC3II/LC3I ratio (Figure 5G′). Despite this, it should be noted that after CQ treatment, we detected a tendency to increase in LC3II/LC3I ratio in all AD fibroblasts compared to controls, although not statistically significant (Figure 5G′). This tendency could be due to inhibition of lysosomal degradation causing autophagosome accumulation, as shown by the increased p62/SQSTM1 signal in *APOE 3/4* + *PSEN1* fibroblasts (Figure 4B). However, we cannot rule out that part of the effect is due to CQ activation of non-canonical autophagy that induces LC3 lipidation in single membrane compartments (Jacquin et al., 2017; Fletcher et al., 2018).

### 3.6. *APOE 3/4 + PSEN1* fibroblasts show a higher number and clustering of lysosomes

**FIGURE 6:**
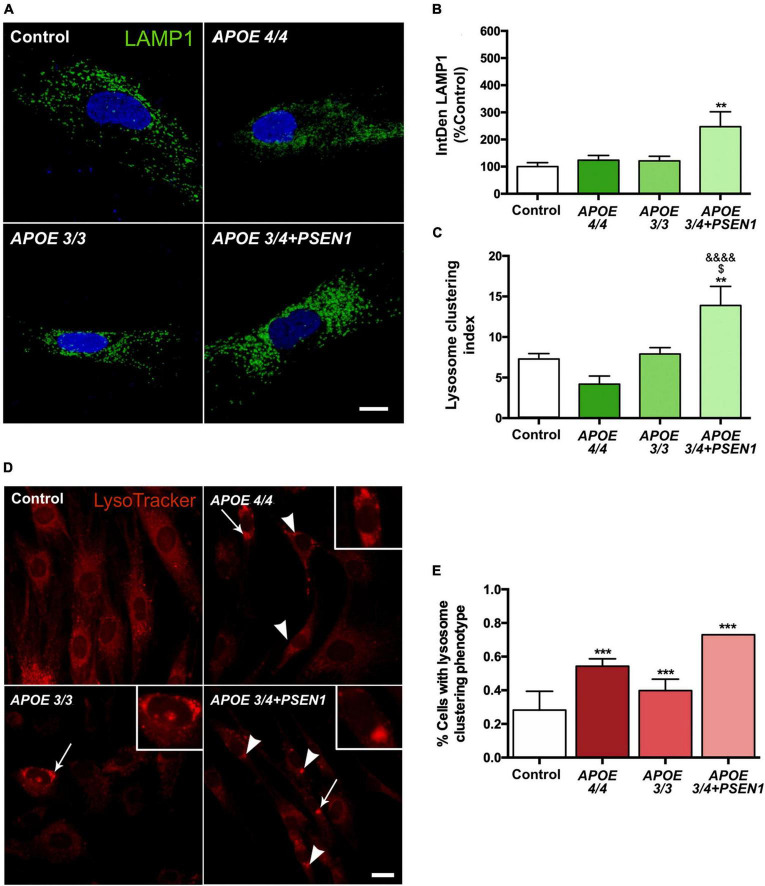
Increased number of lysosomes, lysosomal clustering in *APOE* 3/4 + *PSEN1* fibroblasts, and perinuclear clustered lysosomes in all AD fibroblasts. **(A)** Representative images of LAMP1 (a lysosome marker) immunofluorescence in AD fibroblasts (*APOE* 4/4, *APOE* 3/3 and *APOE* 3/4 + *PSEN1*). **(B)** Integrated density (IntDen = intensity*μm2) of LAMP1. Kruskal-Wallis, *post-hoc* Dunn. **(C)** Lysosomal clustering index (number of clustered lysosomes / number of individual lysosomes). Samples are processed in parallel; data represent mean ± SEM of *n* = 6 images for all cell lines. **P < 0.01 vs. Control; ^$^*P* < 0.05 vs. *APOE* 3/3; ^&&&&^*P* < 0.0001 vs. *APOE* 4/4, 1-way ANOVA, *post hoc* Bonferroni. Calibration bar = 10 μm. **(D)** Representative images of LysoTracker staining. Arrows indicate the zoomed areas in the top right corner of each image. Arrowheads indicate lysosomal clusterings. **(E)** The proportion of fibroblasts of each genotype presenting lysosomal aggregates. Samples are processed in parallel; data represent mean ± SEM of a minimum of 80 cells per genotype analyzed. ***P < 0.005 vs. Control, χ^2^ test. Calibration bar = 20 μm.

To determine whether the increase in autophagy induction contributes to further degradation of the mitochondria, we analyzed the final stage of the pathway, specifically, the distribution and number of lysosomes. We measured LAMP1 (a marker of these organelles), detecting its levels by immunofluorescence (Figure 6A). The lysosomal distribution was assessed by measuring the lysosomal clustering index. We observed that *APOE 3/4* + *PSEN1* fibroblasts present a significant increase not only in the number of lysosomes (Figure 6B) but also in their lysosomal clustering index (Figure 6C). Strikingly, the number and clustering levels of lysosomes in *APOE* 4/4 and *APOE* 3/3 fibroblasts are similar to controls (Figures 6B, C). However, using the LysoTracker probe to label the lysosomes, we found that AD lysosomes were significantly more clustered around the perinuclear region than controls (Figures 6D, E), probably indicating some lysosomal disturbance. Notably, this increase of perinuclear clustered lysosomes is potentiated in *APOE* 3/4 + *PSEN1* fibroblasts.

## 4. Discussion

**FIGURE 7:**
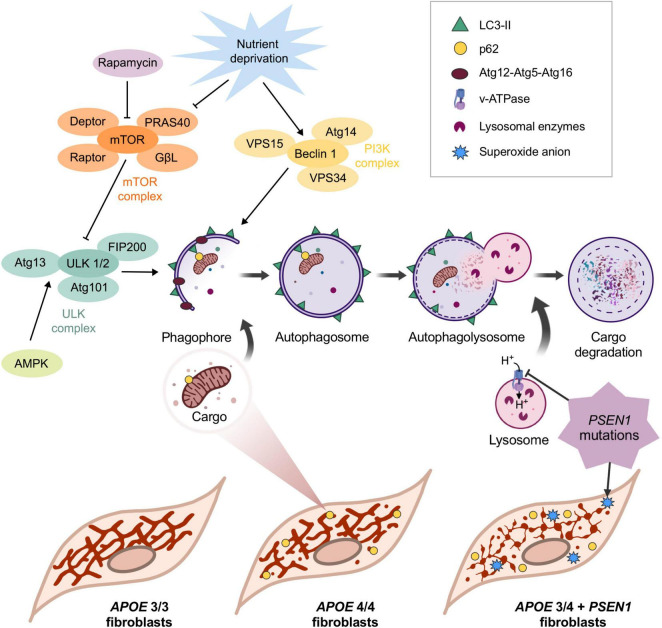
Schematic representation of the main findings of this study. *APOE* allele ε4 in homozygosis and *APOE 3/4* + *PSEN1* induces the fragmentation of the mitochondrial networks. The increase in p62/SQSTM1 in APOE 4/4 and its colocalization with mitochondria indicates that, in this case, the observed loss of mitochondrial biomass may be caused by its degradation via autophagy. Autophagy is first initiated when vesicles come from the plasma membrane, and different organelles fuse to form a double membrane structure called phagophore. The phagophore eventually fuses its two ends surrounding a portion of the cytoplasm that contains material destined to be degraded, generating the autophagosome. The specificity of the process is given by the p62/SQSTM1 complex, which binds to ubiquitinated proteins and is recognized by LC3-II in the inner face of the autophagosome, which recruits the cargo and internalizes it (Johansen and Lamark, 2011). The next step is the maturation and acidification of the autophagosome (Vegh et al., 2019), which fuses with the lysosome, where the hydrolases degrade the cargo (Kimura et al., 2007). This process can only occur if a sufficiently acidic pH is maintained inside the lysosome (Menzies et al., 2015). Rapamycin and nutrient deprivation inhibit the mTOR complex, the primary autophagy inhibitor. In this situation, mTOR stops phosphorylating ULK1 in Ser-757 (this phosphorylation makes ULK1 catalytically inactive), allowing AMPK to phosphorylate it in Ser-317 and Ser-777, activating it (Menzies et al., 2015). On the other hand, under nutrient deprivation, the PI3K class III complex is also activated. The activations of ULK1 and Beclin1 are essential for the formation of the phagophore, which engulfs the charge that will be degraded. After that, the now-called autophagosome fuses with the lysosome to form the autophagolysosome, where the cargo is finally degraded. The mutation in *PSEN1* induces a significant disruption of mitochondrial networks in *APOE 3/4* + *PSEN*1 fibroblasts and an accumulation of lysosomes and higher levels of superoxide anion inside these cells, which are more vulnerable to oxidative stress.

In this study, we evaluated the impact of the ε4 allele of the *APOE* gene and the mutation *G206D* in *PSEN1* on the molecular mechanisms leading to AD pathology. We found that *PSEN1* and *APOE4/4* or *APOE3/4* confer different phenotypes in human fibroblasts, similarly to that reported in iPSC-derived human microglia; however, in microglia, *PSEN1* and *APOE4/4* or *APOE3/4* affects other underlying mechanisms (Konttinen et al., 2019). Our results show alterations in the vulnerability of AD fibroblasts to oxidative stress and a disruption in the mitochondrial network of *APOE* 3/4 + *PSEN1* and *APOE* 4/4 fibroblasts, as summaries in Figure 7. Moreover, the mutation in *PSEN1* also affects the autophagy pathway and the lysosomal function, increasing lysosomal accumulation and clustering along with an increase of global p62/SQSTM1 (Figure 7).

First, we found that *APOE 4/4* and *APOE 3/3* genotypes confer protection to human fibroblasts against oxidative stress-induced cellular vulnerability (Figures 1A–D), which disagrees with the increased ROS described in other AD studies (Pérez et al., 2017; Sarasija et al., 2018; Drabik et al., 2021). This is particularly striking in the case of the *APOE 4/4* genotype because it contributes to mitochondrial respiratory chain disruption (Orr et al., 2019) and impairs mitochondrial neuron function *in vivo* and *in vitro* (Liang et al., 2021). In this regard, a study detected an increase in ROS in the plasma of *APOE 4/4* AD patients (Massaccesi et al., 2019). However, in this case, the differences observed with our results could be due to blood analyses not exhibiting accurate intracellular or tissue ROS content. Moreover, one of the natural mechanisms by which the cell tries to protect itself against oxidative damage is the arrest of the cell cycle to enable the repair of ROS-induced DNA damage. This occurs through the activation of p53, which triggers cell cycle arrest, DNA repair, and activation of apoptosis (Szybińska and Leśniakx, 2017). Fibroblasts from AD patients exhibited ROS-mediated p53 activation, implying that these cells are less susceptible to oxidative stress and, thus, more resistant than control fibroblasts (Naderi et al., 2006; Uberti et al., 2006). This could be why *APOE 4/4* and *APOE 3/3* fibroblasts are less prone to oxidative stress-induced cell death. Moreover, p53-induced antioxidant response *in vivo* could develop a neuroprotective function. However, the neuroprotective effects of p53 in AD remain controversial (Abate et al., 2020).

APOE is an apolipoprotein with an essential function in cholesterol trafficking. The presence of the *APOE* ε4 allele has typically been related to a lipid homeostasis imbalance in AD patients since this isoform has lower transport affinity and binding capacity for lipids and, in particular, cholesterol (Chang et al., 2017; Jeong et al., 2019; Lanfranco et al., 2020). Previous studies with Filipin staining show that AD fibroblasts (Pani et al., 2009) and, more specifically, *APOE 4/4* astrocytes (Lin et al., 2018) present higher levels of free cholesterol compared to controls. Contrary to these, our results do not show evidence of this accumulation (Figures 2A, B). However, we did surprisingly find a remarkable reduction of the free cholesterol levels inside *APOE* 3/4 + *PSEN1* fibroblasts (Figure 2B). This could be due to a defect in its synthesis, which can eventually lead to a disruption in organelle membranes and cell death (King et al., 2016). Therefore, our results could indicate that the observed decreased cell viability of *APOE 3/4* + *PSEN1* fibroblasts could probably be enhanced by this abnormal cholesterol depletion. We also quantified the free cholesterol levels inside lysosomes in our fibroblasts (Figure 2C). Although no significant results were obtained, we observed a slight increment of free cholesterol in *APOE 3/3* and *APOE 4/4* fibroblasts and a slight lessening in *APOE 3/4* + *PSEN1* fibroblasts. Other authors described that the lysosomal accumulation of cholesterol (García-Sanz et al., 2021) could rescue cells from lysosome-dependent cell death (Appelqvist et al., 2011; King et al., 2016). Hence, this lysosomal cholesterol build-up could be one of the reasons why these *APOE* 3/3 and *APOE* 4/4 fibroblasts appear to be protected against cell death. Defects related to lysosome dysregulation, lipid membrane disruption, intracellular cholesterol distribution, and altered Ca^2+^ signaling depend on the *APOE*ε4 allele and sex in immortalized astrocytes (Larramona-Arcas et al., 2020). Therefore, this could be a possible explanation for our *APOE 4/4* results.

Aging stands out as the most pivotal risk factor for neurodegenerative disorders, including AD (Hou et al., 2019). Aging decreases the cellular ability to produce energy (Ozgen et al., 2022; Trigo et al., 2022), and mitochondria play an essential role in producing such energy. They are necessary for regulating critical biochemical processes such as Ca^2+^ storage and homeostasis, activation of the oxidative stress response, and cell death pathways (Ribas et al., 2014). Therefore, mitochondrial dysfunction is closely linked to AD pathogenesis (Swerdlow, 2018; Perez Ortiz and Swerdlow, 2019; Castora et al., 2022). In this context, our results showed that fibroblasts from patients with AD have mitochondria with fewer reticular networks (Figures 3D–I, 5). This is especially evident in *APOE* 3/4 + *PSEN1* fibroblasts, which present dot-shaped mitochondria (Figure 3D). This dotted appearance is observed in other studies as an indicator of fragmented mitochondria in unhealthy and oxidatively stressed cells (Ježek and Plecitá-Hlavatá, 2009).

Moreover, AD fibroblasts, especially those with the ε4 allele of the *APOE* gene in homozygosis, have higher mitochondria labeled with p62/SQSTM1 (Figure 4A), a fragmented mitochondrial network (Figures 3A–E). This could be due to the early enhanced induction of autophagy in AD fibroblasts, as previously described (Bordi et al., 2016). This induction would eventually lead to a higher degradation of damaged mitochondria. In this regard, a slight disruption of the mitochondrial network was detected in all our fibroblasts from AD patients (Figures 3D–I). From the mitochondrial perspective, this could entail a common pathogenic origin of the disease, regardless of the genotype (Yin et al., 2020). Specifically, *APOE 4/4* fibroblasts exhibited increased mitochondrial fragmentation as indicated by the significant increase of individual fragments (Figure 3E) as was shown in other studies (Cabezas-Opazo et al., 2015; Pérez et al., 2017). It was recently determined that despite also showing an impaired mitochondrial network, APOE4 astrocytes displayed an increased number of branches and fewer individual mitochondria, contrary to our findings (Schmukler et al., 2020). Impairments in the mitochondria of these astrocytes are also supported by deficiencies in their synthesis, recruitment, ubiquitination, fission, fusion, and mitophagy (Eran and Ronit, 2022). The mitochondrial disorganization we have found is more evident in the case of *APOE 3/4* + *PSEN1* fibroblasts due to the decreased size of the mitochondrial network, the length of its branches, and the area occupied by mitochondria (Figures 3G–I). This latter parameter, which may also be indicative of the percentage of mitochondrial biomass in the fibroblasts (Sinha et al., 2019; Li et al., 2020), suggests that *APOE 3/4* + *PSEN1* fibroblasts could have a reduction in the overall mitochondrial mass. These mitochondrial abnormalities found in *APOE 3/4* + *PSEN1* fibroblasts agree with those previously found in fibroblasts from AD patients with *PSEN1* mutation (Gray and Quinn, 2015; Bell et al., 2018). This impaired integrity of the mitochondrial network could be responsible for the subtle higher superoxide anion levels and increased vulnerability to oxidative stress of *APOE* 3/4 + *PSEN1* fibroblasts. This mechanism may be primarily caused by the deregulation of the Ca^2+^ homeostasis induced by the mutation in *PSEN1*, as described in *PSEN1* mutant AD iPSC-derived astrocytes (Oksanen et al., 2017). Mitochondria form an interconnected network, which allows them to communicate rapidly and distribute energy throughout the cell (Trigo et al., 2022). However, this connectivity puts the energy conversion system at risk because the entire network could suffer the consequences if any elements are damaged (Trushina et al., 2012). Mitochondria are dynamic organelles constantly fusing and dividing (Bertholet et al., 2016). The dynamic equilibrium between fusion and fission phenomena defines the morphology of the mitochondria, allowing their adaptation to energy needs. Therefore, the increased mitochondrial fragmentation of AD fibroblasts suggests a possible fusion and fission balance disruption. Moreover, previous studies on AD also demonstrated that oxidative stress increases the fragmentation of the mitochondrial network via the deregulation of mitochondrial fusion and fission dynamics (Zhu et al., 2012; Misrani et al., 2021; Olesen et al., 2022).

Another critical mechanism in controlling mitochondrial quality is mitophagy. The defects in mitochondrial networks’ complexity in *APOE 4/4* and *APOE 3/4* + *PSEN1* fibroblasts (Figure 3), aligns with the significantly higher levels of p62/SQSTM1 detected in these fibroblasts (Figure 4B), which probably indicates an induced mitophagy to eliminate these harmful organelles (Figure 7). Consistently, *APOE 4/4* fibroblasts also had a higher percentage of mitochondria labeled with p62/SQSTM1 destined for degradation, as shown by the colocalization analysis (Figure 4C). However, fibroblasts with the *PSEN1* mutation did not show an increased colocalization of their mitochondria with p62/SQSTM1 compared to controls (Figure 4C). These results could be explained because of a defect in mitochondrial biogenesis processes, which would result in aberrant mitochondria, thus, contributing to the pathophysiology of AD, as already demonstrated in previous studies where mitochondrial biogenesis markers such as PGC-1a are reduced (Sheng et al., 2012). Another possibility is that *APOE 3/4* + *PSEN1* fibroblasts undergo an ineffective mitophagy process. This would align with previous studies in *PSEN1* fibroblasts and iPSC-derived neurons from patients, where mitochondria were labeled correctly but unable to be degraded (Trushina et al., 2012). In addition, *APOE 3/3* fibroblasts do not present significant changes in mitochondrial biomass (Figure 3), p62/SQSTM1 levels, and mitochondria labeled with p62/SQSTM1 compared to controls (Figure 4). These results align with the ε3 allele of the *APOE* gene being less related to the pathophysiology of the disease (Heffernan et al., 2016), as also demonstrated in mouse model studies (Simonovitch et al., 2019).

A dampened lysosomal autophagic clearance or an altered activation of autophagy could be responsible for the aforementioned increased p62/SQSTM1 in *APOE 3/4* + *PSEN*1 and *APOE 4/4* fibroblasts (Figure 7). Similarly, higher levels of p62/SQTM1 and parkin were found in the hippocampus of APOE4 mice compared to APOE3 mice, showing reduced mitophagy (Simonovitch et al., 2019). In addition, even though the analysis of the levels of p-mTOR showed no conclusive outcome (Figures 5A, B), in the case of ULK, there was a slight decrease of p-ULK in all AD fibroblasts compared to controls (Figures 5A, C). Beclin1 was also significantly increased in *APOE 3/3* and *APOE 3/4* + *PSEN1* fibroblasts (Figures 5D, E). This is consistent with the increased colocalization of mitochondria with p62/SQSTM1 detected in *APOE 4/4* fibroblasts (Figure 4C), indicating a possible impaired mitophagy. Therefore, these results could indicate that fibroblasts from AD patients may develop an altered and induced autophagic pathway onset, which differs from previous studies with AD patients that show an accumulation of defective mitochondria due to defects in autophagy induction (Martín-Maestro et al., 2017b; Vegh et al., 2019). This could suggest that *PSEN1* mutation might not only accelerate autophagosome synthesis through Beclin1 and mTOR pathways but also alter autophagic clearance (increased p62/SQSTM1) likely due to a secondary degradation defect in lysosomes, triggering an autophagosome buildup (LC3II accumulation after CQ treatment). However, as mentioned before, it cannot rule out that this effect could be due to the non-canonical autophagy activation (Jacquin et al., 2017; Fletcher et al., 2018). In either case, the results could point to an anomalously induced autophagy, probably as a compensatory mechanism due to a decline in the degradation of the last step of autophagy (Martín-Maestro et al., 2017a). Moreover, it must be considered that there is still debate about whether autophagy is altered in single or multiple stages in AD (Bordi et al., 2016), and further studies regarding this are still needed to clarify this question.

Consistently with the possible degradation failure, we found lysosomal impairment in AD fibroblasts. We found higher levels of LAMP1 in *APOE 3/4* + *PSEN1* fibroblasts (Figures 6A, B) which aligns with previous studies displaying elevated LAMP1 levels and its mRNA in AD patients’ cortexes (Barrachina et al., 2006). *PSEN1* mutations generate a defect in the N-glycosylation of the V0a1 subunit of the v-ATPase, causing problems in its transport toward the lysosomes. This fact leads to defects in the acidification of the lysosomes, as well as deficiencies in their proteolysis (Lee et al., 2010; Vegh et al., 2019). Also, under acute mitochondrial stress conditions, AMP-dependent protein kinase (AMPK) is repressed, leading to an accumulation of lysosomal Ca^2+^ and a loss of lysosomal hydrolysis due to defects in acidification (Deus et al., 2020). Thus, the accumulation of lysosomes in our *PSEN1* fibroblasts could be due to these acidification deficiencies. This would result in dysfunctional lysosomes that tend to accumulate, increasing their clustering rate (Figure 6C), as previously described in APP and *PSEN1* mutant neurons (Hung and Livesey, 2018).

Furthermore, the higher proportion of perinuclear lysosome clusters in all our AD fibroblasts (Figures 6D, E), even more, enhanced in those with the *APOE 3/4* + *PSEN1* genotype could also be an indicator of lysosomal disturbance, probably by blocking the lysosomal exocytosis. The cellular distribution of lysosomes is relevant in modulating lysosomal function and coordinating cellular responses to the presence or absence of nutrients (Tancini et al., 2020). One of the cellular responses coordinated by the changes in the intracellular localization of lysosomes is the process of autophagy since mTOR, the primary regulator of this pathway, is found inside lysosomes. The position of lysosomes within the cell changes in response to nutrient availability, thus coordinating the mTOR activity and the successive autophagy induction. When nutrients are scarce, there is an increase in the intracellular pH, and lysosomes move toward the perinuclear region. This causes the inactivation of mTOR, which activates autophagy, facilitating the fusion of the autophagosome with the lysosome. Conversely, when nutrients are available, cytoplasmic pH decreases, lysosomes return to peripheral regions, and mTOR is activated again, thus inhibiting autophagy (Korolchuk et al., 2011). Consequently, AD-derived alterations in this lysosomal transport could be responsible for this perinuclear clustering phenotype, as already described (Kanaan et al., 2013; Hwang et al., 2019; Lie and Nixon, 2019).

In brief, in this study, we have assessed the impact of the ε4 allele of the *APOE* gene and a mutation in *PSEN1(G206D)* on the cellular mechanisms underlying the pathogenesis of AD using skin fibroblasts derived from AD patients (Figure 7). Although this cellular model has some limitations, this experimental approach has allowed us to obtain significant differences between control and patient-derived fibroblasts in several parameters. Hence, fibroblasts can be a good model for studying pathological mechanisms in AD, since they constitute an easily accessible patient-specific cellular model of the disease. This is due to the cellular plasticity of skin fibroblasts, which endows them with the potential to be easily cultured and have levels of gene expression and damage accumulation similar to those of neurons (Bell et al., 2018; Tong et al., 2022).

## 5. Conclusion

Alzheimer’s disease, as in other neurodegenerative diseases, has a systemic element that can affect peripheral cells outside the nervous system, characterized by a series of changes at the metabolic level, such as alterations in autophagy or mitochondrial dysfunction. Therefore, the study of these changes in fibroblasts derived from AD patients can contribute to the deciphering of the molecular physiopathology of the disease. We found that the *APOE* allele ε4 in homozygosis produces an increased fragmentation of the mitochondrial network, probably due to slightly induced mitophagy to eliminate these damaged mitochondria. Moreover, *PSEN1* mutation disrupts the integrity of the mitochondrial network, triggering high superoxide anion levels and, thus, making *APOE 3/4* + *PSEN1* fibroblasts more vulnerable to cell death induced by oxidative stress. In this regard, *G206D-PSEN1* mutation probably produces an autophagosome accumulation due to degradation defect. It induces a buildup and altered distribution of lysosomes, along with an increase of global p62/SQSTM1 that could compromise lysosomal degradation, as shown in Figure 7. All these alterations could contribute eventually to the neuronal degeneration that underlies the pathogenesis of Alzheimer’s disease. However, a limitation of our study is that the *PSEN1* study is based on a single fibroblast cell line, and therefore, the conclusion drawn cannot be generalized; nevertheless, it opens the possibility of having mutation-specific treatments in the future.

This work constitutes an interesting characterization of the mitochondrial status and autophagy mechanisms in patients’ fibroblasts that could offer new targets for developing AD biomarkers and therapies.

## Data availability statement

The raw data supporting the conclusions of this article will be made available by the authors, without undue reservation.

## Ethics statement

The studies involving human participants were reviewed and approved by the Human Research Ethics Committees of CSIC and CIBERNED (Instituto de Salud Carlos III). The patients/participants provided their written informed consent to participate in this study.

## Author contributions

PG-S and RM were responsible for all aspects of the project, including the conceptualization and design of the study. CV provided fibroblasts of AD patients. IC-L, PG-S, and EJ-E performed experiments and acquired and analyzed data. IC-L and PG-S provided the first draft, prepared figures, and discussed the results. PG-S, IC-L, EJ-E, CV, and RM edited the draft. PG-S provided the definitive version. RM and CV provided financial support. All authors revised the final version of the manuscript and took responsibility for its content.
